# Strongyloides stercoralis Infection in Immunocompromised Host

**DOI:** 10.7759/cureus.21217

**Published:** 2022-01-13

**Authors:** Neha Sharma, Kaveh Zivari, Daria Yunina, Matthew Grunwald, Omar Azar, Rabin Rahmani, Kevin Tin

**Affiliations:** 1 Internal Medicine, Maimonides Medical Center, New York City, USA; 2 Gastroenterology, Maimonides Medical Center, Brooklyn, USA

**Keywords:** duodenitis, immunocompromised host, eosinophalia, hyperinfection, helminth, strongyloides

## Abstract

*Strongyloides stercoralis* is a soil-dwelling roundworm that causes an intestinal infection, Strongyloidiasis. In the United States, this helminth generally causes chronic asymptomatic infection, and severe symptomatic infections are reported in immunocompromised hosts like patients undergoing chemotherapy, receiving long-term corticosteroids, transplant patients, or patients with HIV. The clinicians should have a high index of suspicion to diagnose this infection, as the exposure is usually remote and symptoms are non-specific. The treatment is simple, with oral anti-helminthic drugs like ivermectin and albendazole.

## Introduction

*Strongyloides stercoralis* is a soil-dwelling roundworm that causes an intestinal infection in humans, termed Strongyloidiasis, with a global prevalence estimated to be between 30 and 100 million. This helminth is endemic in tropical and subtropical regions of the world, Kentucky and the rural Appalachia region of the United States, and largely causes a chronic, asymptomatic infection [1,2]. Severe symptomatic infections are reported in immunocompromised hosts like patients undergoing chemotherapy, receiving long-term corticosteroids, transplant patients, or patients with HIV [3]. The phenomenon of auto-infection and hyperinfection helps the pathogen to propagate and seed in different tissues and cause fulminant infection, which can be fatal. The clinicians should have a high index of suspicion to diagnose this infection, as the exposure is usually remote and symptoms are non-specific. The diagnosis is difficult due to the absence of a gold standard test, low parasite load, and intermittent larval shedding [4]. The treatment is simple, with oral anti-helminthic drugs like ivermectin and albendazole.

## Case presentation

**Figure 1:**
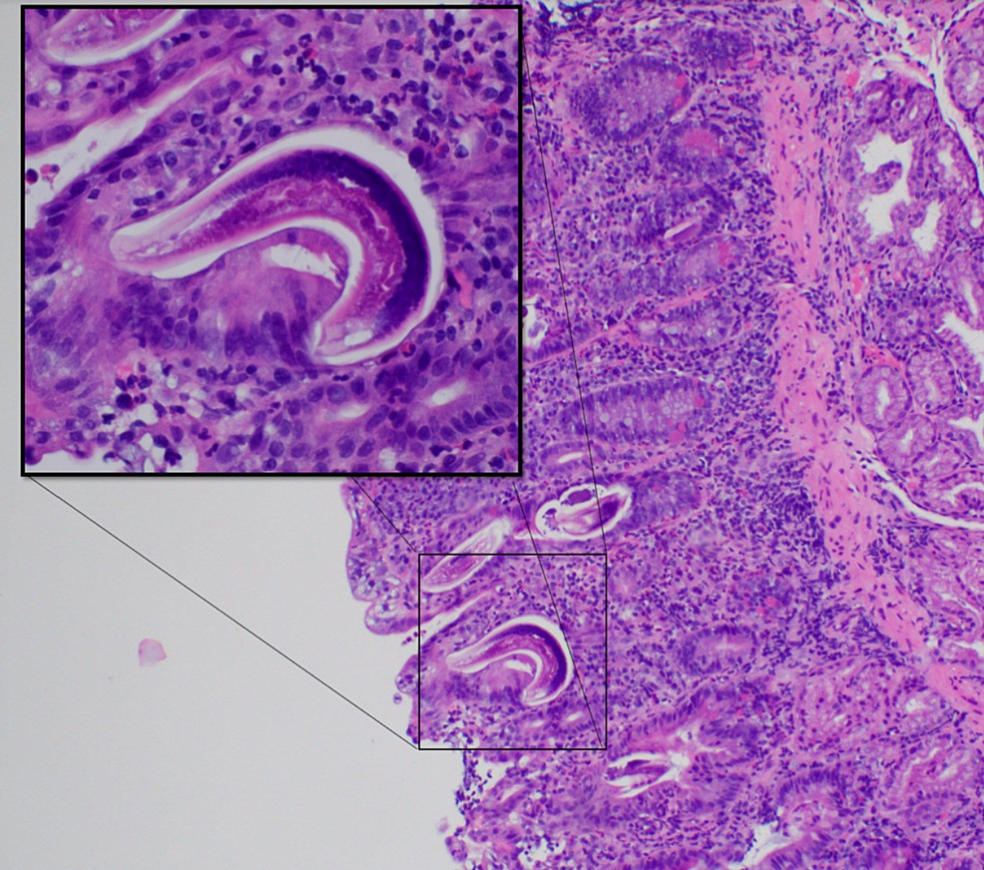
Duodenal mucosa with acute inflammation, reactive changes, and parasitic organisms, which was found to be Strongyloides stercoralis.

A 79-year-old Chinese migrant man with a history of metastatic lung adenocarcinoma, who was started on erlotinib 10 days prior, presented with nausea, vomiting, diarrhea, generalized weakness, and epigastric pain. His CBC revealed WBC 7.6 with 6.6% eosinophils. X-ray and computed tomography imaging of his chest/abdomen/pelvis did not show any changes from prior imaging. Esophagogastroduodenoscopy was performed, showing duodenal ulcer, gastritis and duodenitis. Subsequently, biopsies of the duodenal and esophageal mucosa revealed parasitic larvae consistent with *S. stercoralis *(Figure 1). The patient was started on two days of ivermectin, with a repeat of the therapy in two weeks. The patient had complete resolution of his symptoms and was restarted on erlotinib after discharge. During follow-up three months later, the complete serology for *Strongyloides *was negative.

## Discussion

The biology of *S. stercoralis* involves two separate life cycles: a free-living cycle and a parasitic cycle [5]. The infective larval form enters the human host by penetrating intact skin, and it travels up the bloodstream to reach pulmonary circulation. In the lungs, the larva penetrates the alveoli, ascends the tracheobronchial tree, and is swallowed to reach the intestine where the female worms penetrate the intestinal mucosa and lay the eggs parthenogenetically. These eggs mature into rhabditiform larva to be passed out in feces or re-infect the host through the perianal skin or intestinal mucosa and start the cycle all over again [6,7]. This auto-infective cycle helps the worm to maintain infection in the host indefinitely. In immunocompromised hosts, the rate of autoinfection escapes control by the host, overwhelming the immune system, resulting in hyperinfection and potentially life-threatening infection with up to 85%-100% mortality rate [1,2]. The clinical manifestations of acute infection are associated with larval migration route from a portal of entry to the intestine in the form of skin irritation, dry cough, and eventually gastrointestinal symptoms of diarrhea and abdominal pain. Chronic infection is usually asymptomatic in an immunocompetent host, with laboratory results showing peripheral eosinophilia. Hyperinfection in immunocompromised hosts is characterized by larval penetration beyond the usual route of migration. Patients experience crampy abdominal pain, watery diarrhea, difficulty swallowing, sore throat, bloating along with protein-losing enteropathy, which adds to the debility of the infection [1,4].

Differential diagnosis in a patient with cancer who presents with esophagitis and duodenitis includes radiation-induced inflammation, chemotherapy leading to mucositis, infections such as *Candida*, herpes simplex, enterovirus, bacteria such as *H. pylori*, or parasitic infection. Our patient was diagnosed with esophagitis and duodenitis secondary to *Strongyloides *after tissue biopsy revealed larvae embedded in the intestinal mucosa. The larvae of *Strongyloides *have the potential ability to invade and survive for extended periods of time in human tissue. Depressed cell-mediated immunity secondary to a malignant tumor, combined with protein-calorie malnutrition, leads to a lack of granulomatous immune response to larvae in our patient. Several immunodiagnostic assays have been found ineffective in detecting disseminated *Strongyloides *infections. Although it is important to detect latent *S. stercoralis* infections before administering chemotherapy, a specific and sensitive diagnostic test is lacking. When acute esophagitis and duodenitis does present in these patients, it may be severe and disabling. It might result in hospitalization, placement of a feeding tube, or intravenous feedings. Other feared complications associated with the hyperinfection syndrome include intestinal obstruction due to worm burden or mesenteric lymphadenopathy and Gram-negative sepsis due to burrowing of the larva carrying the enteric microorganisms into the bloodstream [1]. Biopsy, cytology, and culture are recommended to establish the diagnosis.

All the patients, regardless of the severity of the disease, need to be treated. The treatment options include ivermectin or albendazole, with cure rates reaching up to 94%-100% [4].

## Conclusions

*S. stercoralis *infection in a cancer patient is a potentially life-threatening infection, with mortality rates reaching up to 85%-100%. The treatment is fairly uncomplicated with oral antiparasitic drugs like ivermectin or albendazole. Due to high mortality associated with the infection in cancer patients because of lack of immune response and protein-calorie malnutrition, the worms propagate rampantly and cause lethal consequences if not diagnosed and treated, hence it is imperative to screen and treat the patients for Strongyloidiasis prior to commencing the chemotherapy.
